# Usefulness of core needle biopsy of thyroid for the diagnosis of IgG4 Hashimoto's thyroiditis

**DOI:** 10.1515/jtim-2026-0037

**Published:** 2026-03-26

**Authors:** Chenxu Zhao, Yang Yu, Jumei Liu, Yang Zhang, Lei Chen, Guizhi Lu, Ying Gao

**Affiliations:** Department of Endocrinology, Peking University First Hospital, Beijing, China; Department of Pathology, Peking University First Hospital, Beijing, China; Department of Ultrasound, Peking University First Hospital, Beijing, China

**Keywords:** hashimoto's thyroiditis, immunoglobulin G4, core needle biopsy, diagnosis

## Abstract

**Background and Objective:**

IgG4-related Hashimoto's thyroiditis (IgG4 HT) is characterized by rapid progression and may be associated with an increased risk of papillary thyroid carcinoma (PTC). The diagnosis of IgG4 HT relies primarily on postoperative pathological analysis. Early identification of IgG4 HT is crucial for guiding patient management. This study assessed the possibility of thyroid core needle biopsy (CNB) in diagnosing IgG4 HT.

**Methods:**

One hundred and twenty HT patients who underwent color Doppler-guided CNB and subsequent thyroid surgery were collected in Peking University First Hospital. Clinical, serological, sonographic, and histopathological features were also collected. The numbers of IgG4 and IgG plasma cells were counted in five high power fields (HPF), then the average numbers of IgG4+ and IgG+ plasma cells per HPF were calculated respectively.

**Results:**

Based on the IgG4 and IgG immunohistochemistry results of 120 surgical specimens, cases were subclassified as IgG4 HT (*n* = 18) and non-IgG4 HT (*n* = 102) groups by the thyroid-specific diagnostic criteria (IgG4+ plasma cells > 20/HPF and IgG4+/IgG+ plasma cell ratio > 30%). CNB samples from IgG4 HT patients were subsequently subjected to IgG4/IgG immunostaining. However, only eight of the corresponding CNB tissues met the IgG4 HT diagnostic criteria. The remaining ten patients had IgG4+ positivity ranged in 10–20 cells/HPF and an IgG4+/IgG+ plasma cell ratio ranging from 20% to 67%. Histopathological characteristics of thyroid tissue were consistent between the surgical and CNB samples.

**Conclusion:**

IgG4/IgG immunostaining of CNB samples derived from thyroid tissue may serve as a valuable tool for supporting the diagnosis of IgG4 HT.

## Introduction

Hashimoto’s thyroiditis (HT) is an organ-specific autoimmune disease, the annual incidence of which is estimated to be 0.3 to 1.5 cases per 1000 persons.^[1]^ The progression of HT varies significantly among different patients.^[2]^ Once diagnosed, HT patients require lifelong monitoring of thyroid function and should receive thyroxine substitution therapy when hypothyroidism develops.

Immunoglobulin G4-related HT (IgG4 HT) was first reported in 2009.^[3]^ Studies utilizing surgical specimen databases have reported that the prevalence of IgG4 HT among all HT patients ranges from 12.6% to 53.0%.^[4, 5, 6, 7, 8]^ Compared to non-IgG4 HT, IgG4 HT is associated with a greater degree of lymphoplasmacytic infiltration, interfollicular fibrosis and follicular cell degeneration in the thyroid.^[7,9]^ Previous research revealed other specific clinical features associated with IgG4 HT, including younger age, a relatively higher proportion of male patients, higher serum antithyroglobulin IgG4 and anti-thyroperoxidase IgG4 levels, and a more rapid progression to hypothyroidism in IgG4 HT patients.^[5,6]^ However, no significant differences have been observed in serum concentrations of IgG4 or the IgG4/IgG ratio were found between patients with IgG4 HT and non-IgG4 HT.^[5, 10]^ Furthermore, IgG4 HT is associated with an increased risk of papillary thyroid carcinoma (PTC), and patients with both IgG4 HT and PTC tend to have adverse prognoses.^[5, 8]^ Therefore, early detection and diagnosis of IgG4 HT are important.

The current diagnostic criteria for IgG4 HT are as follows ^[11, 12]^ : (1) meeting the criteria of HT, which mainly included lymphocytic infiltration of the stroma, a germinal center formation, oxyphilic change of the follicular epithelium, Hurthle cell change, follicular destruction, and variable degrees of fibrosis ^[13, 14]^ ; (2) dense infiltration of lymphocytes and plasma cells, with IgG4-positive plasma cells > 20/ HPF and a high IgG4/IgG-positive plasma cell ratio of > 30%; and (3) stromal fibrosis. Patients with other diseases that exhibit elevated tissue levels of IgG4, such as primary thyroid lymphoma, are excluded.^[12]^ These criteria and cutoffs are based on studies of surgical resection samples from HT patients.

While this approach provides accurate histopathologyical diagnosis, it is of limited utility in preoperative clinical diagnoses and treating patients. A well-established method for the preoperative diagnosis of IgG4 HT might enable efficient management and treatment of this disease. Given the limited size of tissue obtained through biopsy samples, distinct diagnostic standards may be required.

Notably, there continues to be debate on the diagnostic standards of IgG4 related disease (IgG4-RD) for specific sites, such as the pituitary gland, necessitating further research and refinement.^[9]^ A previous study revealed the diagnostic value of biopsy samples in IgG4-RD, due to limited tissue volume of core needle biopsy (CNB) samples, different cutoff values of IgG4+ plasma cell are applied compared to those used for surgical samples.^[15, 16, 17]^ Whether the diagnostic criteria based on surgical samples are appropriate for the diagnosis of IgG4 HT using biopsy specimens is worth exploring.

Thyroid endoscopic ultrasound (US)-guided CNB is an established technique for evaluating thyroid masses. Unlike fine-needle aspiration, CNB samples contain not only the lesion but also adjacent normal thyroid tissue, which can be used for pathological examination. Jin *et al*. diagnosed three clinically suspected patients with IgG4 HT by performing IgG4 and IgG immunostaining of CNB samples.^[18]^ Interestingly, despite steady progress regarding the clinical, ultrasonic, and pathologic characteristics of IgG4 HT, comprehensive studies examining the role of CNB in its diagnosis are lacking.

We examined 120 HT patients who underwent US-guided CNB and subsequent thyroid surgery at Peking University First Hospital. By performing IgG4 and IgG immunohistochemical staining and analyzing pathological characteristics, we evaluated the efficacy of CNB for the diagnosis of IgG4 HT and explored the possibility of establishing criteria for diagnosing IgG4 HT on the basis of CNB specimens.

## Materials and methods

### Patients

The data of 236 patients with a histopathologically confirmed diagnosis of HT who were treated at Peking University First Hospital from April 2009 through January 2021 were collected retrospectively. All these patients underwent thyroid surgery due to thyroid cancer or compressive symptoms. They had undergone preoperative CNB, therefore, data from fine needle aspiration (FNA) were not available. Clinical information, including age, sex, and thyroid functional status, was obtained from the patients’ medical records. Furthermore, the clinical records were meticulously assessed to determine the presence of any possible indicators or manifestations of extrathyroidal IgG4-related disease. All pathological sections were independently reassessed by two pathologists. Exclusion criteria were the absence or too small of an HT background in the CNB biopsy samples (*n* = 85), or with focal tumor-associated lymphocytic thyroiditis (diffuse infiltration of lymphocytes and other inflammatory cells, no typical histopathological features of HT) (*n* = 4), or other coexisting thyroid diseases in thyroid tissue (*n* = 27). Ultimately, a total of 120 HT patients were included in the study. This study complied with the Helsinki Declaration and was approved by the Ethics Committee of Peking University First Hospital (Approval No. KY2018-09). The requirement for informed consent from the patients was waived owing to the retrospective study design.

### Laboratory evaluation

Serum thyrotropin (TSH), free thyroxine (FT4), total thyroxine (TT4), free triiodothyronine (FT3) and total triiodothyronine (TT3) levels were measured using a chemiluminescent immunoassay (ADVIA Centaur, Siemens, Germany). The TgAb and TPOAb levels were determined through an electrochemiluminescent immunoassay (Cobas601, Roche Diagnostics, USA).

### Us-guided CNB procedure

Ultrasonography was performed with conventional grayscale and color Doppler using 5–10 MHz linear transducers (LA523 probe, Esaote, Genova, Italy). Thyroid biopsy was performed by experienced sonographers under ultrasonic guidance using an 18-gauge transfixion pin of a Bard automatic biopsy device (BARD, Bard International Inc., Covington, GA, USA). Avoiding important structures such as blood vessels, trachea, and nerves, the end of the biopsy needle was advanced into the edge of the thyroid nodule of interest, and the stylet and cutting cannula of the needle were sequentially fired. The target nodule tissue, nodule-parenchyma borders (and/or visible capsules), and normal thyroid parenchyma are usually obtained, as suggested in prior studies.^[19, 20, 21, 22]^ For hyperechoic HT nodules with enlarged thyroid glands accompanied by compression symptoms, the thyroid parenchyma from the side with more significant disease involvement was selected. Two to three core needle biopsy tissues were obtained from each HT patient. No complications related to CNB were observed in any of the 120 patients during the procedures or follow-up.

During the CNB procedure, peripheral tissues surrounding the nodule were also collected. CNB specimens with an HT background, collected more than 1 cm away from cancerous areas and confirmed to be cancer-free by pathologists, were used for analysis. For patients with nodular goiter, thyroid adenomas or adenomatous hyperplasia, CNB or surgical samples were collected from areas distant from any pathological nodules. In HT patients with thyroid cancer, surgical samples from the thyroid lobe ipsilateral to the biopsy site and as far away from cancerous foci as possible were collected from each patient and subsequently selected for staining. All selected tissue samples were then used for staining and analysis. As a subtype of HT, IgG4 HT is also pathologically characterized by diffuse lymphocytic infiltration in the thyroid glands. Thus, although the CNB and surgical tissues were not from the same anatomic region of the thyroid, both could be used in the diagnosis of IgG4 HT.

### Histological evaluation and IgG/IgG4 immunohistochemistry of the surgical samples

Thyroid tissue samples from surgically resected specimens were stained with hematoxylin and eosin (H & E). The following histological features of each sample, including the infiltration of lymphocytes and plasma cells, interstitial fibrosis, follicular cell degeneration, and giant cell/ histiocyte infiltration, were graded from negative to 3+ as described in previous studies.^[4, 7]^

Antibodies against IgG (rabbit polyclonal, A0423, 1: 8000, Dako Cytomation, Glostrup, Denmark) and IgG4 (mouse monoclonal, HP6025, 1: 500, Southern Biotech, Birmingham AL, USA) were applied to serial paraffin sections for IgG and IgG4 immunohistochemistry. IgG+ and IgG4+ cells were counted and averaged in five different high-power fields (HPFs) (Olympus BX51T microscope, magnification 400), which were selected on the basis of the highest density of positive cells.

Two pathologists independently reviewed the above features. According to the literature, the histological criteria for diagnosis of IgG4 HT were as follows ^[4,11]^: (1) the fibroinflammatory process remains restricted within the thyroid gland, without any spread beyond its capsule or impact on surrounding structures; (2) immunostaining cutoffs, counting only plasma cells outside of germinal centers: IgG4+ plasma cells > 20/HPF and an IgG4+/ IgG+ cell ratio > 30%; (3) stromal fibrosis. Of the 120 HT patients, 18 patients were diagnosed with IgG4 HT.

### Histopathological assessment of CNB specimens from patients with IgG4 HT

Thyroid tissue samples obtained *via* CNB from all 18 IgG4 HT patients who were surgically diagnosed were subjected to IgG and IgG4 immunohistochemical staining. The number of IgG+ and IgG4+ cells per HPF was counted and quantified as described above.

### Statistical analysis

Statistical analyses were conducted *via* the SPSS 26.0 software package (SPSS Inc.). For normally distributed data, the results are presented as the arithmetic mean ± SD. Data exhibiting a skewed distribution were summarized using the median and interquartile range. Paired data of CNB and surgical specimens were analysed by the Wilcoxon matched-pairs test. *P* values less than 0.05 were considered statistically significant.

## Results

### Clinical characteristics

Table 1 shows the basic characteristics of the 120 HT patients. The mean age of the HT patients was 45.6 ± 12.3 years (range, 14–79 years) at the time of surgery, and the majority (85%) were female. Postoperative pathology revealed HT complicated with PTC (*n* = 112), benign adenoma (*n* = 3), nodular goiter (*n* = 3), hyalinizing trabecular tumor (*n* = 1), and adenomatous hyperplasia (*n* = 1). Among the 120 patients, 5 patients had hypothyroidism and were treated with L-thyroxine, 4 patients had subclinical hypothyroidism, 103 patients were euthyroid, 3 patients had thyroid toxicosis (“Hashitoxicosis”) and were treated with methimazole, and 4 patients had subclinical hyperthyroidism. None of the clinical records revealed any evidence of other IgG4-RD organ involvement

**Table 1:** General characteristics of enrolled 120 Hashimoto’s thyroiditis patients

Characteristics	Values
Age (years)^a^	45.6±12.3
Gender (female/male)	102/8
Disease duration (month)^b,c^	3 (1, 18)
Indication for CNB (suspicious malignant thyroid nodules on US/ compression)	116/4
Extrathyroidal IgG4-RD (+/-)	0/120
TSH (μIU/mL)^b,d^	2.33 (1.53, 2.79)
FT3 (pmol/L)^b,d^	4.67 (4.4, 4.95)
FT4 (pmol/L)^b,d^	14.74 (13.69, 16.00)
TT3 (nmol/L)^b,e^	1.68 (1.50, 1.90)
TT4 (nmol/L)^b,e^	103.6 (89.38, 118.80)
TgAb (IU/mL)^b,f^	157.8 (48.5, 364.5)
TPOAb (IU/mL)^b,f^	35.0 (12.0, 160)
TgAb positive (*n*%)^b,f^	60 (53.6%)
TPOAb positive (*n*%)^b,f^	55 (49.1%)
TgAb or TPOAb positive (*n*%)^b,f^	88 (78.6%)
Thyroid function (hyperthyroidism/ euthyroidism/hypothyroidism)^b,g^	7/103/9

a, Data are the mean ± standard deviation. b, Data are the median (Q1, Q3). c, One case with missing information on disease duration. d, One cases with missing information on TSH, FT3, and FT4. e, Two cases with missing information on TT3 and TT4. f, Eight cases with missing information on TgAb and TPOAb. g, One cases with missing information on thyroid function. IgG4-RD, immunoglobulin G4-related disease; TSH, thyrotropin; FT3, free triiodothyronine; FT4, free thyroxine, TT3, total triiodothyronine; TT4, total thyroxine. TgAb, thyroglobulin autoantibodies; TPOAb, thyroid peroxidase antibodies. Values of <5 and >600 for TPOAb, <20 and >4000 for TgAb, >30 for TSH, were used as 5, 600, 20, 4000, and 30, respectively, in calculations.

### Histological evaluation

**Figure 1:**
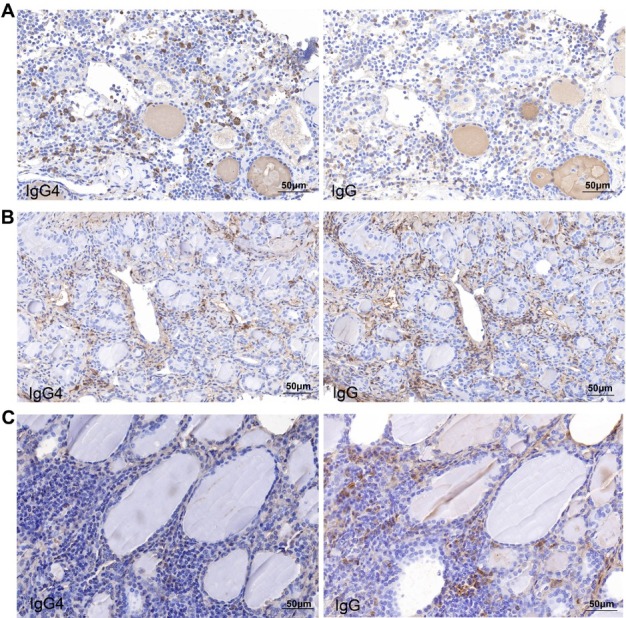
Immunostaining for immunoglobulin (Ig) G4 and IgG in IgG4 HT patients and non-IgG4 HT patients. Serial sections of thyroid surgical tissue (A) and CNB tissue (B) from an IgG4 HT patient. A marked increase in IgG4+ and IgG+ plasma cells in thyroid tissue was detected. (C) Serial sections of thyroid surgical tissue from a non-IgG4 HT patient. IgG4-positive plasma cells are scarce, and there is infiltration of IgG+ plasma cells. Magnification ×400.

Surgical specimens from all 120 HT patients were collected for HE evaluation and IgG4 immunostaining. Using the thyroid-specific diagnostic criteria mentioned above, 18 out of 120 HT patients were categorized as IgG4 HT patients (Figure 1). Unlike previous reports, no differences in the demographic, clinical, serological, or sonographic features were noted between the patients with IgG4 HT versus non-IgG4 HT patients (Supplementary Table S1). This discrepancy might be related to the unavoidable selection bias of the retrospective study designs.

In terms of histopathological features, storiform fibrosis, lymphoplasmacytic infiltration, and giant cell or histiocytic infiltration were detected in the CNB samples from all 18 IgG4 HT patients, whereas the CNB samples from nine patients were insufficient for the assessment of follicular epithelial degeneration and thyroid follicle size. The histopathological details are reported in Table 2. Quantitative analysis demonstrated that the histopathological severity scores for these features were significantly lower in CNB specimens than in corresponding surgical sepcimens (Table 3). This discrepancy may be attributed to sampling bias, as CNB specimens may not fully capture the most severely affected regions of the thyroid due to the patchy distribution of IgG4-positive plasma cells. Additionally, the lack of universally accepted pathological classification for IgG4 HT across studies may contribute to inconsistencies in case identification and group assignment.

**Table 2:** Clinicopathological features in 18 patients diagnosed as lgG4 HT by surgical specimens

Patients		1	2	3	4	5	6	7	8	9	10	11	12	13	14	15	16	17	18
Age (F/M) (year)/Gender		50/F	40/F	59/F	30/F	50/M	52/F	52/F	50/F	34/F	20/F	53/F	40/F	30/M	29/F	36/F	49/F	51 /F	61 /F
TSH (*μ*lU/mL)		1.07	2.74	4.72	0.5	3.35	1.64	5.63	1.43	1.89	1.17	1.82	0.9	1.55	2.08	2.25	0.77	1.47	0.05
FT3 (pmol/L)		4.65	5.29	5.1	6.13	4.72	3.47	5.1 1	4.94	4.86	5.58	4.48	5.31	4.72	4.77	4.91	3.02	5	7.15
FT4 (pmol/L)		16.95	18.64	14.59	19.76	15.84	13.54	17.16	14.74	14.92	13.58	15.31	17.51	13.64	13.2	15.86	13.62	17.12	16.17
TT3 (nmol/L)		2.08	2.07	2.03	1.54	1.72	1.3	1.4	1.56	2.09	1.85	1.55	1.85	1.62	1.51	1.83	0.99	2.02	-
TT4 (nmol/L)		147.2	143.5	105.2	99.3	90.1	93.5	1 17.1	102	126	93.2	83.2	1 15.4	92.7	102.6	125.2	97.9	123	-
TgAb (lU/mL)		1214	1 17.1	32.55	24.19	78.91	252.7	153.4	92.51	292.8	18.7	>4000	2510	264.8	513.7	345.8	45.2	22.02	93.2
TPOAb (lU/mL)		19.43	16.87	51.24	6.06	133	9.59	22.84	15.87	66.84	14.54	595.9	44.8	**<5**	139.4	139.7	8.91	5.83	35.36
lgG4 + plasma cells (n/HPF)	Surgical specimen	22	42	54	48	25	69	32	28	57	70	**60**	43	53	33	57	20	58	40
	CNB specimen	12	22	17	44	10	32	14	18	10	20	50	22	40	30	17	12	35	25
lgG4 + /IgG + ratio (%)	Surgical specimen	32	49	55	53	30	45	58	36	67	35	83	20	56	71	67	30	42	52
	CNB specimen	20	33	20	52	40	37	33	28	20	16	55	28	36	47	41	18	30	40
Stromal fibrosis degree	Surgical specimen	3	2	3	2	3	1	3	3	1	1	1	3	1	1	3	3	2	1
	CNB specimen	3	1	3	2	1	0	2	1	1	1	1	3	1	0	1	3	1	1
Lymphoplasmacytic infiltration	Surgical specimen	2	3	2	3	3	3	3	3	3	3	2	2	2	2	3	3	3	3
	CNB specimen	2	2	2	2	3	3	2	2	3	3	1	1	2	2	3	2	3	2
Giant cell or histiocytic infiltration	Surgical specimen	1	3	3	3	2	2	3	3	2	1	**0**	0	1	1	3	2	3	2
	CNB specimen	1	0	3	2	1	0	2	3	2	1	**0**	0	1	1	3	2	3	2
Thyroid follicle size (micro-, 1; normo-or macrofollicle, 0)	Surgical specimen	0	1	1	1	1	1	1	1	0	0	1	1	0	0	0	1	1	0
	CNB specimen	NA	NA	1	NA	0	NA	1	1	NA	NA	1	1	0	NA	NA	1	1	NA
Follicular epithelial degeneration	Surgical specimen	2	3	3	3	2	2	3	1	2	3	3	3	2	1	1	2	3	3
	CNB specimen	NA	NA	3	NA	1	NA	2	3	NA	NA	3	2	1	NA	NA	2	3	NA

NA, the tissue was not available for analysis. TSH, thyrotropin; FT3, free triiodothyronine; FT4, free thyroxine, TT3, total triiodothyronine; TT4, total thyroxine. TgAb, thyroglobulin autoantibodies; TPOAb, thyroid peroxidase antibodies. CNB, core needle biopsy.

**Table 3:** Comparison of pathological characteristics between surgical and core needle biopsy specimens of thyroid tissues from 18 patients with IgG4 Hashimoto’s thyroiditis

Pathological characteristics	IgG4 Hashimoto’s thyroiditis		*P* value
	Surgical specimens	Core needle biopsy specimens	
Stromal fibrosis degree (3+/2+/1+/-)	8/8/2/0	5/6/5/2	0.009
Lymphoplasmacytic infiltration (3+/2+/1+/-)	10/8/0/0	6/10/2/0	0.005
Giant cell or histiocytic infiltration (3+/2+/1+/-)	7/5/4/2	4/5/5/4	0.039
Thyroid follicle size (micro-/normo-or macrofollicle)	11/7	7/2, 9NA	0.010
Follicular epithelial degeneration (3+/2+/1+/-)	9/6/3/0	4/3/2/0, 9NA	0.480

NA, the tissue was not available for analysis.

### Rate of detection of IgG4 HT with CNB using diagnostic criteria for surgical specimens

**Figure 2:**
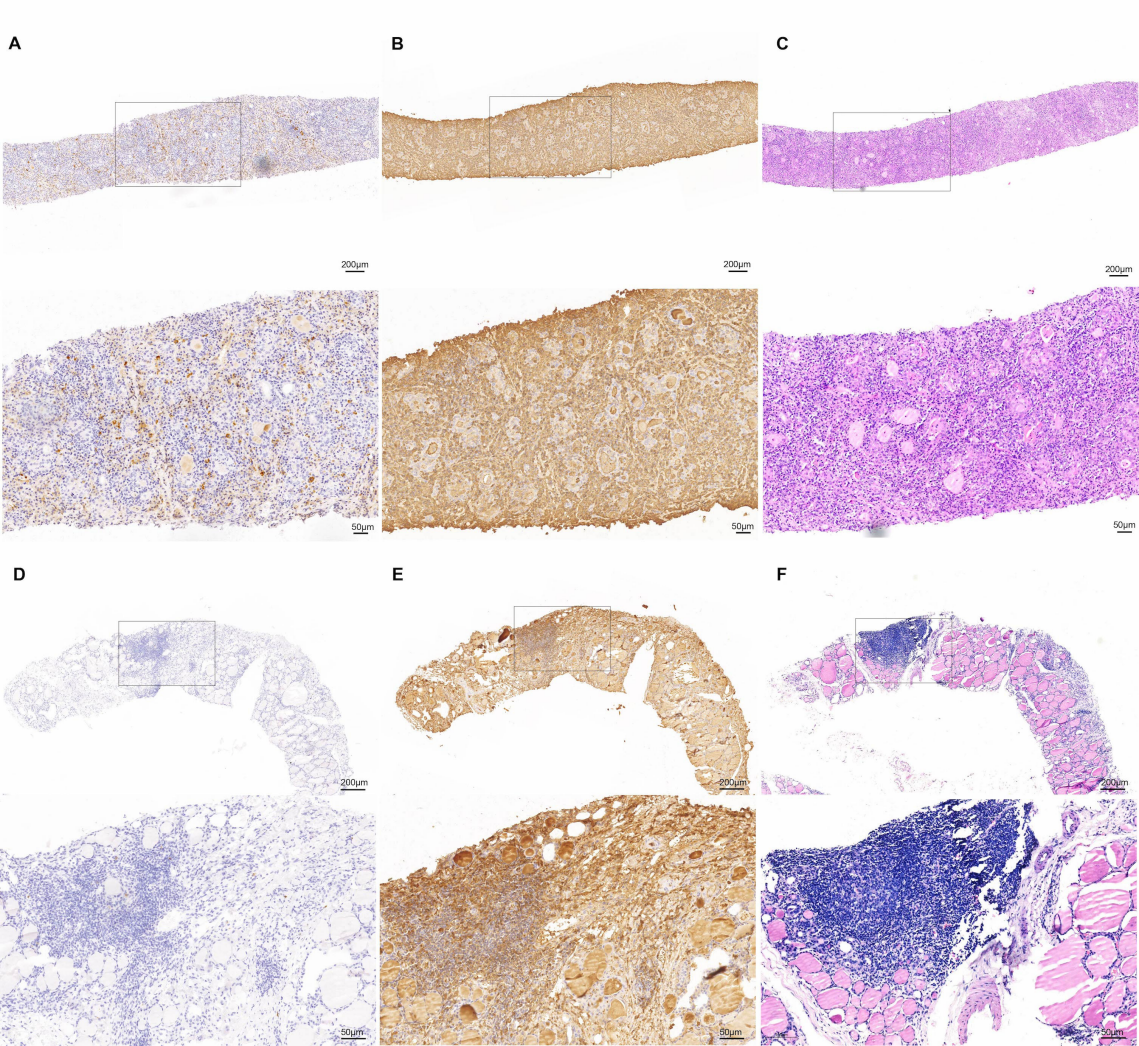
Immunohistochemical staining of immunoglobulin (Ig) G4 and IgG in core needle biopsy (CNB) samples from two IgG4 HT patients. Markedly increased IgG4+ positive plasma cells (a) and IgG+ positive plasma cells (b) were detected in the CNB specimen of Patient 6. However, in the CNB specimen from Patient 17, a low number of IgG4+ positive plasma cells (d) and extensive IgG+ positive plasma cells (e) were observed. Histopathologically, both patients presented higher grades of stromal fibrosis, lymphoplasmacytic infiltration, and follicular cell degeneration (c, f).

As shown in Table 3, among the CNB samples from 18 IgG4 HT patients, only 8 (8/18; 44.4%) indicated a diagnosis of IgG4 HT (Figure 2a–c). Fewer IgG4+ plasma cells (range, 10–20/HPF) and lower IgG4+/IgG+ plasma cell ratios (range, 20%–30%) were detected in the CNB samples from the remaining ten patients (Figure 2d–f) (Table 3). These ten patients could not be diagnosed with IgG4 HT because the quantitative assessment of IgG4+ plasma cells and the IgG4+/IgG+ plasma cell ratio did not reach the cutoff point for IgG4 HT (> 20 IgG4+ plasma cells per HPF and a > 30% IgG4/IgG+ plasma cell ratio).

## Discussion

Compared with non-IgG4 HT, IgG4 HT results in pronounced lymphoplasmacytic infiltration, dense fibrosis, and notable follicular cell degeneration.^[3,7]^ In addition to these histopathological characteristics, subsequent studies have highlighted discernible clinical and ultrasonographic distinctions between these two conditions. Patients with IgG4 HT are typically younger, have a higher male-to-female ratio, exhibit more diffuse low echogenicity on ultrasound, and experience a more aggressive course with a higher incidence of subclinical hypothyroidism compared to those with non-IgG4 HT.^[5,7,18]^ Therefore, early diagnosis could mitigate the need for unnecessary surgery and subsequent complications. Unexpectedly, no differences in the clinical or ultrasonographic characteristics were noted between the IgG4 HT group and the non-IgG4 HT group in this study. This might have been due to the selection bias, as HT patients who underwent surgery and concurrent CNB were included. In addition, CNB samples lacking sufficient HT background tissues were excluded.

Previously, we devised a noninvasive diagnostic model utilizing machine learning techniques to identify high-risk IgG4 HT patients on the basis of TgAb IgG4 and TPOAb IgG4 serum levels.^[23]^ However, these diagnoses of IgG4 HT still need to be confirmed by pathological examination. Sufficient tissue for histopathological and immunohistochemical analysis is essential for the accurate diagnosis of IgG4 HT. IgG4 HT is currently diagnosed by surgical specimens. US-guided CNB, a minimally invasive and accurate procedure,^[19,24]^ has demonstrated high diagnostic performance in the histological diagnosis of many diseases, ^[16, 25,26,27]^ including IgG4-RD.^[15, 28, 29]^ In previous case report of a case in which IgG4 HT was diagnosed by CNB specimens, however, a definitive diagnosis on the basis of CNB is suggested when thyroidectomy is not indicated.

To our knowledge, our study is the first to evaluate the diagnostic performance of CNB-based pathology for IgG4 HT in a cohort study. Based on a preliminary analysis of 120 HT patients who underwent US-guided CNB and subsequent thyroid surgery, we demonstrated the safety and feasibility of this procedure for diagnosing IgG4 HT. CNB samples exhibited lower severity scores for histopathological features compared to surgical samples, which was due to the sampling limitations of CNB, as fragmented biopsy material might not capture the most severely affected tissue areas. However, the consistent identification of histopathological features across 18 patients with IgG4 HT reinforces the diagnostic utility of CNB in IgG4 HT. Notably, all CNB samples from the thyroids of 18 patients with IgG4 HT were sufficient for evaluating the three main criteria required for a final histopathological diagnosis. These findings suggest that CNB specimens are reliable and practical alternatives for diagnosing IgG4 HT, particularly when thyroidectomy is not warranted.

It also remains unclear whether or not cutoff value for diagnosing IgG4 HT on CNB specimens is the same as that on surgical specimens. Our prospective cohort study revealed a greater prevalence of IgG4 HT in surgical samples than in CNB samples.^[23]^ In this study, we performed IgG/IgG4 immunostaining in CNB specimens from 18 IgG4 HT patients. Of these, only 44.4% of patients met the surgical specimen-based diagnostic criteria using CNB samples. Similar results were reported in other IgG4-related diseases, such as IgG4-related sclerosing cholangitis and autoimmune pancreatitis,^[30, 31]^ in which biopsy samples presented notably lower counts of both IgG+ and IgG4+ plasma cells compared to surgical samples.^[32]^ In some IgG4-RD cases, such as IgG4-related pancreatitis, IgG4+ plasma cells > 50/HPF and an IgG4+/IgG+ plasma cell ratio > 40% have been proposed as histological diagnostic criteria for diagnosis on the basis of surgical specimens.^[15]^

For biopsy samples, the presence of 10 IgG4+ plasma cells per HPF has been reported to be highly specific for diagnosis.^[17]^ The different cutoff values of IgG4+ plasma cells between surgical and biopsy samples are related to the patchy distribution of IgG4+ plasma cells in tissues.^[15, 32]^ Our results, combined with previous observations, suggest a lower cutoff point of IgG4+ plasma cells for diagnosis of IgG4 HT; however, further research is needed to better define the optimal cutoff value.

This study has several limitations. First, it is a retrospective diagnostic analysis based on preoperative CNB samples from patients who were diagnosed with HT histopathologically. Additionally, because this is a retrospective study and serum IgG4 data were not available, we were unable to perform a direct comparison between serological and histopathological findings. Furthermore, HT patients with combined thyroid tumors were not excluded from our study. The tumor-related inflammation could potentially influence IgG4+ plasma cell infiltration, we specifically evaluated tissues collected at least 1 cm away from the cancerous lesion to minimize peritumoral effects.^[33, 34]^ Thirdly, the present study included a small number of IgG4 HT patients and CNB specimens from non-IgG4 HT patients were not collected, thus it was not possible to perform a receiver operating characteristic curve analysis to determine optimal diagnostic thresholds specific to CNB. Therefore, further research that include both IgG4 HT and non-IgG4 HT cases with corresponding CNB samples is needed to establish the utility of thyroid biopsies in distinguishing between IgG4-HT and non-IgG4 HT.

The present study indicates that CNB of the thyroid is useful for the diagnosis of IgG4 HT and shows significant promise for further clinical application. Notably, a lower cutoff point of IgG4+ plasma cells and IgG4+/IgG+ ratio for may improve the accuracy for diagnosis of IgG4 HT based on CNB specimens. However, the diagnostic cutoff value for IgG4 HT in thyroid CNB specimens needs to be tested with a larger number of patients in a prospective study. The use of thyroid-specific classification criteria for IgG4 HT could improve disease subtyping and enable more targeted treatment. Ultimately, CNB can help avoid unnecessary surgeries and refine diagnostic thresholds, improving patient outcomes.

## Supplementary Material

Supplementary Material Details
