# Supplementary material for: Usefulness of core needle biopsy of thyroid for the diagnosis of IgG4 Hashimoto's thyroiditis
Source: J Transl Int Med. 2026 Mar 26;14(2):306–14. doi: 10.1515/jtim-2026-0037 (PMC13110463; doi:10.1515/jtim-2026-0037)
Supplement: Supplementary file 1 — Supplementary Material Details [file jtim-2026-0037_sm.pdf]

Supplementary materials

Supplementary Table S1: Comparison of clinical findings between IgG4 HT and non-IgG4 HT

	IgG4 HT (*n* = 18)	Non-IgG4 HT (*n* = 102)	*P*
**Age (years)**	43.7 ± 11.7	45.9 ± 12.5	0.843
**Gender (F/M)**	16/2	96/6	0.414
**Disease duration (mo)** [a]	5 (3, 12)	3 (1, 18.25)	0.365
**Sonographic features (normal echogenicity/hypoechogenicity/ heterogeneous echogenicity)** [a]	10/0/8	37/1/59	0.184
**Indication for thyroidectomy (sPTC/NL/other)**	14/3/1	93/6/3	0.098
**Concurrent PTC (+/-)**	16/2	96/6	0.414
**TgAb (IU/mL)** [b]	167 (51.3, 366)	181 (52.9, 366)	0.974
**TPOAb (IU/mL)** [b]	35 (12, 159.5)	41.1 (12.2, 180.1)	0.257
**Thyroid function (hyperthyroidism/ euthyroidism/hypothyroidism)**	1/15/2	6/88/7	0.625

a, Missing information;

b, Values of <5, <20, >30, >600, and >4000 were used in calculations as 5, 10, 30.0, 600, and 4000, respectively.
